# Croup as Unusual Presentation of Post-transplantation Lymphoproliferative Disorder after Liver Transplantation in an 18-month-old Child

**Published:** 2016-02-01

**Authors:** A. Keshtkari, S. M. Dehghani, M. Haghighat, M. H. Imanieh, A. Nasimfard, G. Yousefi, H. Javaherizadeh

**Affiliations:** 1 Social Determinants of Health Research Center, Yasuj University of Medical Sciences and Department of Pediatric Gastroenterology, Shiraz University of Medical Sciences, Shiraz, Iran,; 2 Shiraz Transplant Research Center, Gastroenterohepatology Research Center, Nemazee Teaching Hospital, School of Medicine, Shiraz University of Medical Sciences, Shiraz, Iran,; 3 Department of Pediatric Infectious Disease, Nemazee Hospital, Shiraz University of Medical Sciences, Shiraz, Iran,; 4 *Department of Pediatric Gastroenterology, Nemzaee Hospital, Shiraz University of Medical Sciences, Shiraz, Iran*

**Keywords:** Lymphoproliferative disorders, Liver transplantation, Immunosuppression, Tacrolimus, Rituximab, Prednisolone

## Abstract

Post-transplantation lymphoproliferative disorder (PTLD) is a serious complication of solid organ transplantation that occurs due to immunosuppression and other risk factors. PTLD may present with involvement of other organs and with unusual presentation. The presentation is often extranodal (*e.g.*, in the gastrointestinal tract, lung, or the central nervous system). Herein, we report on a 1.5-year-old girl who underwent liver transplantation almost 5 months prior to admission. She was on medications such as tacrolimus and prednisolone. Her presentation was started with symptoms of the upper respiratory infection followed by croupy cough and respiratory distress with no response to usual treatments. She had respiratory arrest during broncoscopy. Therefore, emergency tracheostomy was done. Biopsy from the paratracheal mass revealed large B cell non-Hodgkin lymphoma (PTLD, monomorphic and high grade). This case presentation shows that persistent upper airway symptoms, particularly stridor and croupy cough, in children who underwent liver transplant should be further evaluated; the physician needs to have a high degree of clinical suspicion for the diagnosis of PTLD in this situation.

## INTRODUCTION

Post-transplantation lymphoproliferative disorder (PTLD) is a known and serious complication of solid organ transplantation, such as liver transplantation, that occurs as a result of immunosuppression. The occurrence of PTLD depends on the age of the patient, the severity of immunosuppression, Epstein-Barr virus (EBV) status of the patient and the donor, type of organ transplantation, and other risk factors [1]. PTLD involvement is mostly frequent in intestinal or multiorgan transplantion (11%–33%), however, in liver transplantation it ranges from 1%–3%. The lowest PTLD incidence (nearly 1%) occurs in renal transplants [2]. Newell, *et al*, described that the intensity of immunosuppression is a major risk factor for development of PTLD [3]. Cyclosporine and tacrolimus are often used as primary immunosuppression. These drugs were associated with development of PTLD in 4.3% and 6.6% of cases [3]. The clinical presentation of PTLD is variable: Fever, weight loss, and fatigue resembling those seen in infectious mononucleosis are common. Lymphadenopathy, malfunction of the involved organ and symptom of compression effect are other common presentations [1].

## CASE REPORT

A 1.5-year-old girl, a case of cirrhosis due to biliary atresia who underwent liver transplantation five months before was referred for prolonged rhinorrhea, fever, croupy cough and progressive respiratory distress since a few days before her admission. In physical examination, she had low-grade fever, tachypnea, nasal flaring and intercostal retraction without wheezing and lymphadenopathy. A complete blood count and cell differentiation were normal. Serum ALT and AST levels were 28 and 20 U/L, respectively. She had a CRP of 95 mg/L, ESR of 68 mm/h, and LDH of 716 IU/L.

She used sirulimus (1 mg/day po), prednisolone (5 mg/day po), and tacrolimus (2 mg/day po bid).

**Figure 1:**
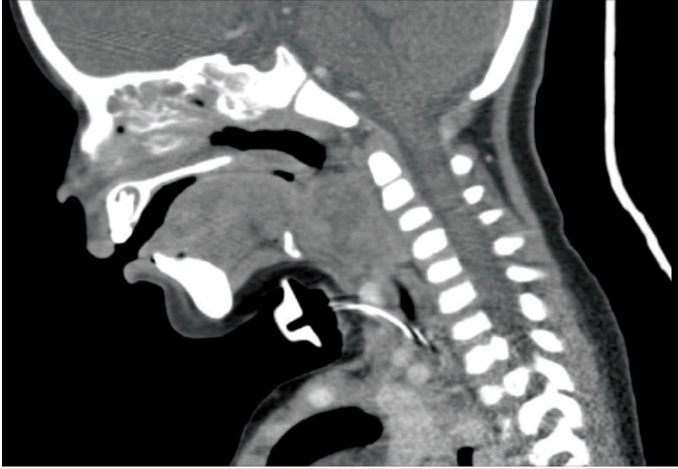
Heterogenous enhancing mass lesion measuring 33×27 mm seen at the level of the epiglottis in the midline and right paramedial aspect with pressure effect over airways leading to airway obstruction

From the first day of admission, with impression of laryngotracheobronchitis, management of croup was started, but no improvement achieved. Therefore, broad-spectrum antibiotics (vancomycin-meropenem) were added and bronchoscopy was planned due to an uncertain history of foreign body aspiration. Respiratory arrest occurred during the induction of anesthesia before bronchoscopy. Resuscitation and attempt for orotracheal intubation failed due to edematous larynx and pharynx. Then, emergency tracheostomy was done without any other investigations. An emergency spiral neck computed tomography showed a heterogenous enhancing mass lesion sized 33×27 mm at the level of the epiglottitis in the midline and right paramedial aspect with pressure effect over airways leading to airway obstruction (Fig 1). Biopsy from the lesion depicted non-Hodgkin large B cell lymphoma. 

With diagnosis of PTLD (monomorphic, high grade), rituximab (375 mg/m^2^), and gancyclovir were stated and change in the immunosuppressive regimen (tacrolimus: from 2 mg bid changed to 1 mg bid, then to 1 mg qd; sirolimus 1 mg/day po) was made. She was transferred to Oncology Ward for further chemotherapy.

**Figure 2:**
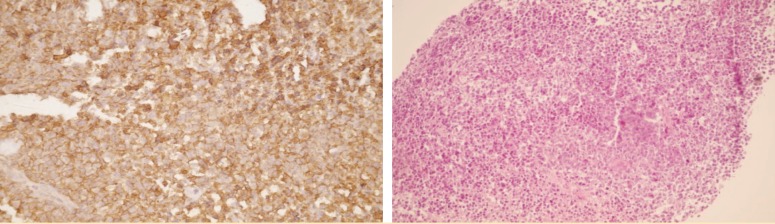
Immunohistochemical staining showing large B-cell lymphoma: Cells are positive for CD20, CD43, and CD79, and are negative for CD3

Immunohistochemistry revealed that cells were positive for CD20, CD43, and CD79, and were negative for CD3 (Fig 2).

Bone marrow aspiration was normal. PCR was negative for HSV and CMV. EBV antigenemia (1000 copy/mL) was detected by quantitative PCR. The patient’s condition improved after the treatment. During follow-up period, the patient expired due to pneumonia unresponsive to medical therapy. 

## DISCUSSION

PTLD is one of the potentially fatal and serious complications of post-solid organ transplantation. PTLD is the most frequent tumor in children following transplantation, occurring in the majority of patients within two years of transplantation [4]. In most patients, it appears due to proliferation of B cell lymphocyte induced by EBV infection associated with immunosuppression [1]. 

Ho, *et al*, described 21 cases of PTLD in 1467 organ transplantation cases with an incidence of 4% in pediatric patients (11 of 253 children transplanted) and 0.8% in adults [5]. Geramizadeh, *et al*, reported that the incidence of PTLD in our center is 0.9%, with 60% mortality rate and worse prognosis in the pediatric age group [6]. 

One of the major risk factors for developing PTLD is immunosuppression. Whiteside, *et al*, revealed that FK506 and cyclosporine-A have profound effects on lymphocyte kinetics and function [7]. 

Manifestations of PTLD are variable: Fevers, lymphadenopathy, anorexia, weight loss, chronic diarrhea, and gastrointestinal blood loss are among the described manifestations. Most of the manifestations of PTLD are present in the gastrointestinal tract that may be the only site of involvement. The disease can cause acute perforation and a surgical abdomen, but it is usually insidious. A patient presenting with this symptom should be evaluated to rule out PTLD. Although extra-gastrointestinal manifestations of PTLDs (*e.g.*, involvement of the lung, or the central nervous system) are uncommon, they make the diagnosis very difficult. Definite diagnosis of PTLD is important. Early diagnosis of this potentially lethal condition is paramount so that treatment can be promptly instituted. To predict the development of PTLD, EBV titers have been widely used. Heslop described that the measurement of EBV load by quantitative PCR can be a sensitive test for the diagnosis, but it is not always specific for predicting the disease onset [8]. Nevertheless, Lee, *et al*, described that the specificity and positive predictive value of EBV titers in predicting PTLD are poor [9]. 

Our patient had unusual presentation of PTLD. She presented with stridor and croupy cough, without lymphadenopathy and with respiratory arrest during intubation about five months after liver transplantation; she was on FK506 and prednisolone (with a plasma level of 28 ng/mL). A decrease in the dose of FK506 and prednisolone and then changing to sirolomus were made. Ho, *et al*, described that the upper airway obstruction was the most common presentation in 9 of 14 cases with PTLD in the Children’s Hospital of Pittsburg; one case had an intratracheal mass with stridor [5]. Hammer, *et al*, described a case of PTLD with severe enlargement of the epiglottis and aryepiglottic fold causing life-threatening airway obstruction after the induction of anesthesia. He presented with progressive stridor. During tonsillectomy, the patient had marked inspiratory stridor, so tracheal intubation was done for the patient. Pathology of the specimens of the epiglottis revealed diffuse B cell hyperplasia [10]. Petti, *et al*, in a review article showed that organ transplant recipients are at risk of oral post-transplantation lymphoproliferative disorders. They described that oral PTLDs usually appear as a solitary swelling, mass, or ulceration, with or without necrotic areas, which is rarely painful, affecting mainly the gingiva, tongue, hard palate, or oropharynx. According to the results of other studies, it is shown that oral PTLDs are rare [11]. 

In conclusion, this case presentation shows that persistent upper airway symptoms, particularly stridor and croupy cough, in children who had undergone liver transplantation need further evaluation; upper airway computed tomography and other investigations are recommended to rule out PTLD. This may establish the diagnosis of PTLD as soon as possible in its course and may decrease morbidity and mortality.
